# MiR-134-3p targets HMOX1 to inhibit ferroptosis in granulosa cells of sheep follicles

**DOI:** 10.1186/s13048-023-01328-6

**Published:** 2024-01-02

**Authors:** Gulimire Abudureyimu, Yangsheng Wu, Ying Chen, Liqin Wang, Geng Hao, Jianguo Yu, Jianguo Wang, Jiapeng Lin, Juncheng Huang

**Affiliations:** 1 Key Laboratory of Genetics, Breeding and Reproduction of Grass-Feeding Livestock, Ministry of Agriculture (MOA), Urumqi, 830026 Xinjiang China; 2 Key Laboratory of Animal Biotechnology of Xinjiang, Urumqi, 830026 Xinjiang China; 3 Institute of AnimalBiotechnology, Xinjiang Academy of Animal Science, Urumqi, 830026 Xinjiang China; 4 Institute of Animal Sciences, Xinjiang Academy of Animal Science, Urumqi, 830000 Xinjiang China

**Keywords:** Sheep, Granulosa cells, Ferroptosis, Oar-miR-134-3p

## Abstract

**Background:**

The intricate interplay of gene expression within ovarian granulosa cells (GCs) is not fully understood. This study aimed to investigate the miRNA regulatory mechanisms of ferroptosis during the process of follicle development in lamb GCs.

**Methods:**

Employing transcriptome sequencing, we compared differentially expressed mRNAs (DE-mRNAs) and miRNAs (DE-miRNAs) in GCs from lambs treated with follicle-stimulating hormone (FL) to untreated controls (CL). We further screened differentially expressed ferroptosis-related genes and identified potential miRNA regulatory factors. The expression patterns of HMOX1 and miRNAs in GCs were validated using qRT‒PCR and Western blotting. Additionally, we investigated the regulatory effect of oar-miR-134-3p on HMOX1 and its function in ferroptosis through cell transfection and erastin treatment.

**Results:**

We identified a total of 4,184 DE-mRNAs and 304 DE-miRNAs. The DE-mRNAs were mainly enriched in ferroptosis, insulin resistance, and the cell cycle. Specifically, we focused on the differential expression of ferroptosis-related genes. Notably, the ferroptosis-related genes HMOX1 and SLC3A2, modulated by DE-miRNAs, were markedly suppressed in FLs. Experimental validation revealed that HMOX1 was significantly downregulated in FL and large follicles, while oar-miR-134-3p was significantly upregulated compared to that in the CLs. HMOX1 expression was regulated by the targeting effect of oar-miR-134-3p. Functional assays further revealed that modulation of oar-miR-134-3p influenced HMOX1 expression and altered cellular responses to ferroptosis induction by erastin.

**Conclusion:**

This study suggested that oar-miR-134-3p and HMOX1 may be one of the pathways regulating ferroptosis in GCs. This finding provides new clues to understanding the development and regulatory process of follicles.

**Supplementary Information:**

The online version contains supplementary material available at 10.1186/s13048-023-01328-6.

## Introduction

Reproductive efficiency in sheep is a cornerstone of effective animal breeding, with the health and functionality of ovarian granulosa cells (GCs) being of paramount importance [1]. Follicle development is a necessary step for ovulation and subsequent fertilization. GCs not only provide nutrition and support for oocytes but also produce hormones that drive follicle development and ovulation [2]. The binding of follicle-stimulating hormone (FSH) to its receptors on GCs is a well-established mechanism that drives these processes [3]. However, the intricacies of how FSH influences GC proliferation and oocyte maturation at the molecular level, particularly through the modulation of microRNA (miRNA) expression, are not fully elucidated.

MiRNAs have emerged as critical post-transcriptional regulators that fine-tune gene expression and are implicated in a myriad of cellular processes, including those pivotal to reproductive biology [4]. Specifically, the crucial role of miRNAs in regulating GC function and ovarian physiology has been widely recognized [5]. In GCs, miRNAs exert their regulatory effects at the post-transcriptional level by specifically binding to target mRNAs, thereby affecting their stability or translation efficiency and regulating gene expression [6]. Despite the recognition of their importance, the specific miRNA‒mRNA interactions and their functional outcomes in the context of FSH signalling remain to be comprehensively mapped.

Adding another layer to this complexity is the process of ferroptosis, a form of programmed cell death characterized by iron-dependent lipid peroxidation, which has been implicated in various biological and pathological states [7, 8]. Studies have found that iron overload-induced ferroptosis in GCs inhibits oocyte maturation [9]. Inhibiting ferroptosis in ovarian GCs using ferroptosis inhibitors can improve ovarian function [10]. However, the role of ferroptosis within the dynamic environment of GCs in lambs, as well as whether miRNAs play a role in regulating GC ferroptosis, remains unanswered.

Therefore, this study aims to bridge these knowledge gaps by deploying high-throughput sequencing to analyse the miRNA and mRNA expression landscapes in lamb GCs in the presence and absence of FSH treatment. By elucidating the miRNA profiles associated with ferroptosis and their target mRNAs, we sought to unravel the molecular tapestry of GC regulation. These data will enhance our understanding of the role of miRNAs in GC function and the role of ferroptosis in ovarian physiology, providing new possibilities for improving reproductive efficiency.

## Materials and methods

### Collection of granulosa cells

Six healthy Merino lambs (aged 6 weeks) were selected from the Sheep Research Center, XinJiang Academy of Animal Sciences to extract follicular GCs. Lambs were treated with or without FSH to obtain two groups: 3 control lambs (CLs) and 3 lambs treated with FSH (FLs). For the FL group, lambs were intraperitoneally injected with 40 IU FSH four times every 12 h. Ovaries were collected under anaesthesia with xylidinothiazoline (2 mg/kg body weight), and follicular fluid was then obtained. GCs were harvested from follicular fluid and centrifuged at 1200 × g for 8 min.

Experimental procedures were carried out in full accordance with the Guide for the Care and Use of Animals of XinJiang Academy of Animal Sciences. All experimental procedures were approved by the Ethics Committee of the XinJiang Academy of Animal Sciences.

### RNA sequencing

Total RNA, including mRNA and miRNA, was extracted from GCs in 3 CLs and 3 FLs using TRIzol Reagent (Invitrogen, USA). The quality and purity of the RNA were assessed using a NanoDrop ND-2000 (Thermo Fisher Scientific, USA) and Agilent 2100 Bioanalyzer (Agilent Technologies). For mRNA sequencing, the NEBNext® Ultra™ Directional RNA Library Prep Kit for Illumina® (NEB, USA) was used to generate the sequencing library, strictly following the manufacturer's instructions. For miRNA, the sequencing library was generated using the NEBNext® Small RNA Library Prep Set for Illumina® (NEB). The constructed libraries were then subjected to high-throughput sequencing on the Illumina HiSeq 2500 platform.

### Differential expression and enrichment analysis

The quality control of sequencing data was performed using the FastQC tool. For mRNAs, reads were aligned to the sheep genome using HISAT2 software. Then, we used featureCounts to count the number of reads for each gene. For miRNAs, reads were aligned to the miRBase database to determine the expression of miRNAs. The analysis of differentially expressed mRNAs (DE-mRNAs) and miRNAs (DE-miRNAs) between CLs and FLs was performed using the DESeq2 software package, with a threshold set at *p* value < 0.05 and |log2FC (fold change)|> 1.

GO (Gene Ontology) and KEGG (Kyoto Encyclopedia of Genes and Genomes) enrichment analyses were performed for DE-mRNAs using the clusterProfiler R package. A *p* value < 0.05 was set as the significance criterion.

### Construction of regulatory networks for miRNAs and mRNAs

Target mRNAs for DE-miRNAs were predicted using the miRanda database [11] and RNAhybrid database [12]. Identification of intersections with differentially expressed ferroptosis-related genes in the predicted miRNA target mRNAs was performed. The regulatory network was constructed based on the regulatory relationships between miRNA and its target mRNAs. The network was visualized using Cytoscape software.

### Culture, transfection and treatment of granulosa cells

GCs were harvested from small follicles (1–2 mm in diameter) and large follicles (≥ 3 mm in diameter) in control lambs. GCs were resuspended in DMEM/F12 culture medium containing 10% foetal bovine serum and 1% antibiotic antifungal agent. Then, GCs were cultured at 37 ℃ and 5% CO_2_ in culture plates containing 2 × 10^5^ cells per well.

The oar-miR-134-3p mimics, inhibitor, and NC (normal control) were purchased from Gemma Medical Technology Co., Ltd. (Shanghai, China). GCs from large follicles were seeded in a 24-well plate and transfected with oar-miR-134-3p mimics (100 nmol/μl), oar-miR-134-3p inhibitor (100 nmol/μl), and oar-miR-134-3p NC (100 nmol/μl) using Lipofectamine 3000 (Invitrogen, Shanghai, China) for 48 h. Subsequently, GCs were treated with or without erastin (ferroptosis inducer; Beyotime, Shanghai, China) for 24 h.

### Cell proliferation analysis

Cells were seeded into a 96-well plate at approximately 5 × 10^3^ cells per well. Then, the cells were further cultured to allow them to attach to the plate. Cell proliferation was assessed using a CCK-8 kit (Cell Counting Kit-8; Beyotime). First, 10% CCK-8 solution was added to each well, and then, the plate was returned to the incubator for an additional 24 h. Absorbance was measured at 450 nm using a microplate reader.

### Dual-luciferase reporter assays

The region of HMOX1's 3'UTR containing the oar-miR-134-3p target site was cloned and inserted into the pmirGLO luciferase miRNA expression reporter vector (Gemma), creating the WT-HMOX1 reporter plasmid. Moreover, we mutated the target site to create the MUT-HMOX1 reporter plasmid. Subsequently, HEK293T cells were seeded into 24-well plates at approximately 5 × 10^4^ cells per well. Once the cells adhered to the well, we transfected them using Lipofectamine™ 2000 (Invitrogen) following the manufacturer's instructions. Each well was transfected with 50 ng of either the WT-HMOX1 or MUT-HMOX1 reporter plasmid and 10 nM oar-miR-134-3p mimic or NC. After 48 h of transfection, we measured luciferase activity using the Dual-Luciferase Reporter Assay System (Promega) according to the manufacturer's instructions.

### ELISAs

Cell culture medium was collected and used to perform ELISAs for MDA and GSH according to the manufacturer's instructions (Biosharp, Wuhan, China).

### Analysis of ROS and mitochondrial membrane potential

GCs were seeded in a 24-well plate and stained with dihydroethidium (DHE, 3 μM, Biosharp), followed by incubation at 37 °C for 20 min. With a fluorescence microscope, red fluorescence was observed in the TRITC channels. The mean red fluorescence was determined as the value of ROS.

GCs were seeded in a 24-well plate and stained with JC-1 dye (5 μM, Biosharp), followed by incubation at 37 °C for 20 min. With a fluorescence microscope, green and red fluorescence were observed under FITC and TRITC channels, respectively. The ratio of red to green fluorescence was determined as the value of the mitochondrial membrane potential.

### qRT‒PCR analysis

Total RNA was extracted from GCs using TRIzol Reagent (Invitrogen). One microgram of total RNA was used for reverse transcription reactions, with the miScript II RT Kit (QIAGEN, China) used for miRNAs and the PrimeScript™ RT reagent Kit (TaKaRa, Japan) for mRNAs, following the manufacturer's instructions. For miRNAs, the miScript SYBR Green PCR Kit (QIAGEN) was used, and for mRNAs, SYBR Premix Ex Taq™ II (TaKaRa) was used to perform qRT‒PCR detection. Each reaction had a total volume of 20 μl, including 10 μl of SYBR Green PCR Master Mix, 0.8 μl of forward and reverse primers (10 μM), 2 μl of cDNA and 7.2 μl of RNase-free water. The qRT‒PCR was run on a StepOnePlus™ Real-Time PCR System (Applied Biosystems), with the reaction conditions set at 95 °C predenaturation for 5 min, followed by 40 cycles of 95 °C denaturation for 10 s and 60 °C annealing and extension for 30 s. Each sample was run with three technical replicates. U6 and B2M were used as the internal reference genes for miRNAs and mRNAs, respectively. The relative expression levels were calculated using the 2^−ΔΔCt^ method. Primers are shown in Table S1.

### Western blot

Proteins were extracted from tissue samples using RIPA protein extraction buffer. The protein concentration was determined using the BCA method. The extracted proteins were mixed with SDS sample buffer and denatured by heating at 100 °C. Equal amounts of denatured protein samples were loaded onto the gel sample slots and subjected to SDS‒PAGE electrophoresis. PVDF was activated by soaking in methanol or transfer buffer. Protein transfer to the membrane was performed using a wet transfer system for 2 h. The membrane was blocked with 5% nonfat milk to prevent nonspecific binding. The membrane was incubated with primary antibodies specific to HMOX1 (ABclonal, Wuhan, China; No. 3522110806; 1:2000) and β-actin (ABclonal; No. 9100; 1:2000) at 4 °C for 8 h. After the membrane was washed, it was incubated with a fluorescent or enzyme-labelled secondary antibody at room temperature for 2 h. The chemiluminescent substrate was used to develop a detectable signal from the labelled secondary antibody (Abcam; No. GR3422216-4; 1:5000). The membrane signal was detected using a chemiluminescence imaging system. The detected signal was quantitatively analysed using ImageJ software (NIH, MD, USA). The signal intensity of HMOX1 was compared to that of the internal reference protein (β-actin) to calculate the relative expression level of HMOX1.

### Statistical analysis

Every experiment was performed with at least three biological replicates, and the data are presented as the means ± standard errors of three replicates. Statistical analysis was performed using GraphPad Prism v.9 (GraphPad, CA, USA). Student’s t test (two-tailed) or one-way analysis of variance was used for comparisons. A *p* value < 0.05 was considered to indicate statistical significance.

## Results

### Identification of DE-mRNAs in GCs

**Fig. 1:**
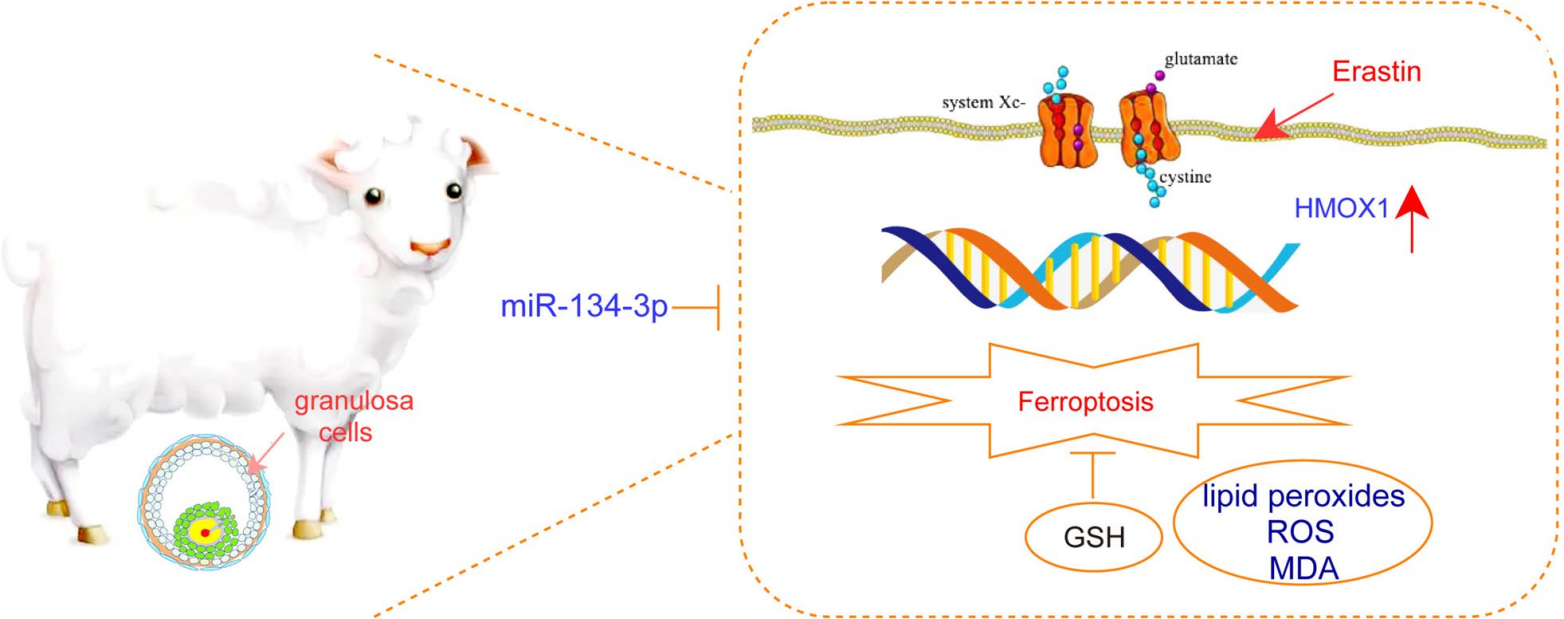
Graphical abstract of this study

**Fig. 2:**
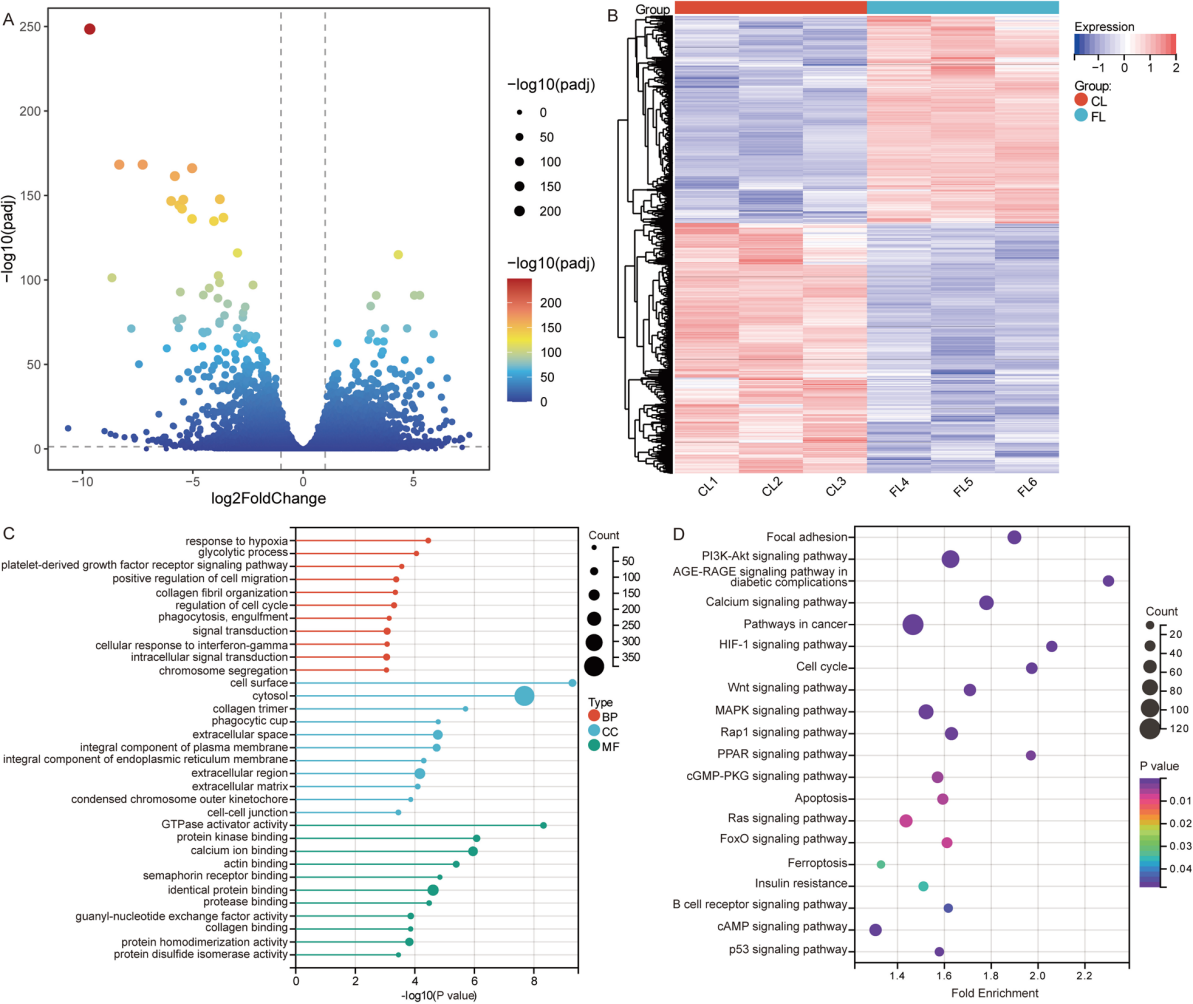
Identification of DE-mRNAs and enrichment analysis in GCs. **A** Volcano map of differentially expressed mRNAs in GCs between CLs and FLs. **B** Heatmap of differentially expressed mRNAs in GCs between CLs and FLs. **C** GO functions of differentially expressed mRNAs in enrichment results. BP, biological processes; CC, cellular components; MF, molecular function. **D** KEGG pathways of differentially expressed mRNAs in the enrichment results

A schematic diagram of this study is shown in Fig. 1. Based on transcriptome sequencing data, we analysed differentially expressed genes in GCs between CLs and FLs. In the results, we identified 4184 DE-mRNAs (Fig. 2A, B). Among them, 1787 mRNAs were significantly higher in FLs than in CLs, while 2397 mRNAs were significantly lower in FLs than in CLs. The enrichment analysis results showed that DE-mRNAs were significantly enriched in response to hypoxia, glycolytic process, and platelet-derived growth factor receptor (GFR) signalling pathway of biological processes (BPs); cell surface, cytosol, and collagen trimer of cellular components (CCs); and GTPase activator activity, protein kinase binding, and calcium ion binding of molecular functions (MFs) (Fig. 2C). In addition, we found that ferroptosis, insulin resistance, and the cell cycle were mainly involved in KEGG pathways (Fig. 2D).

### Regulation of ferroptosis-related genes by miRNAs

**Fig. 3:**
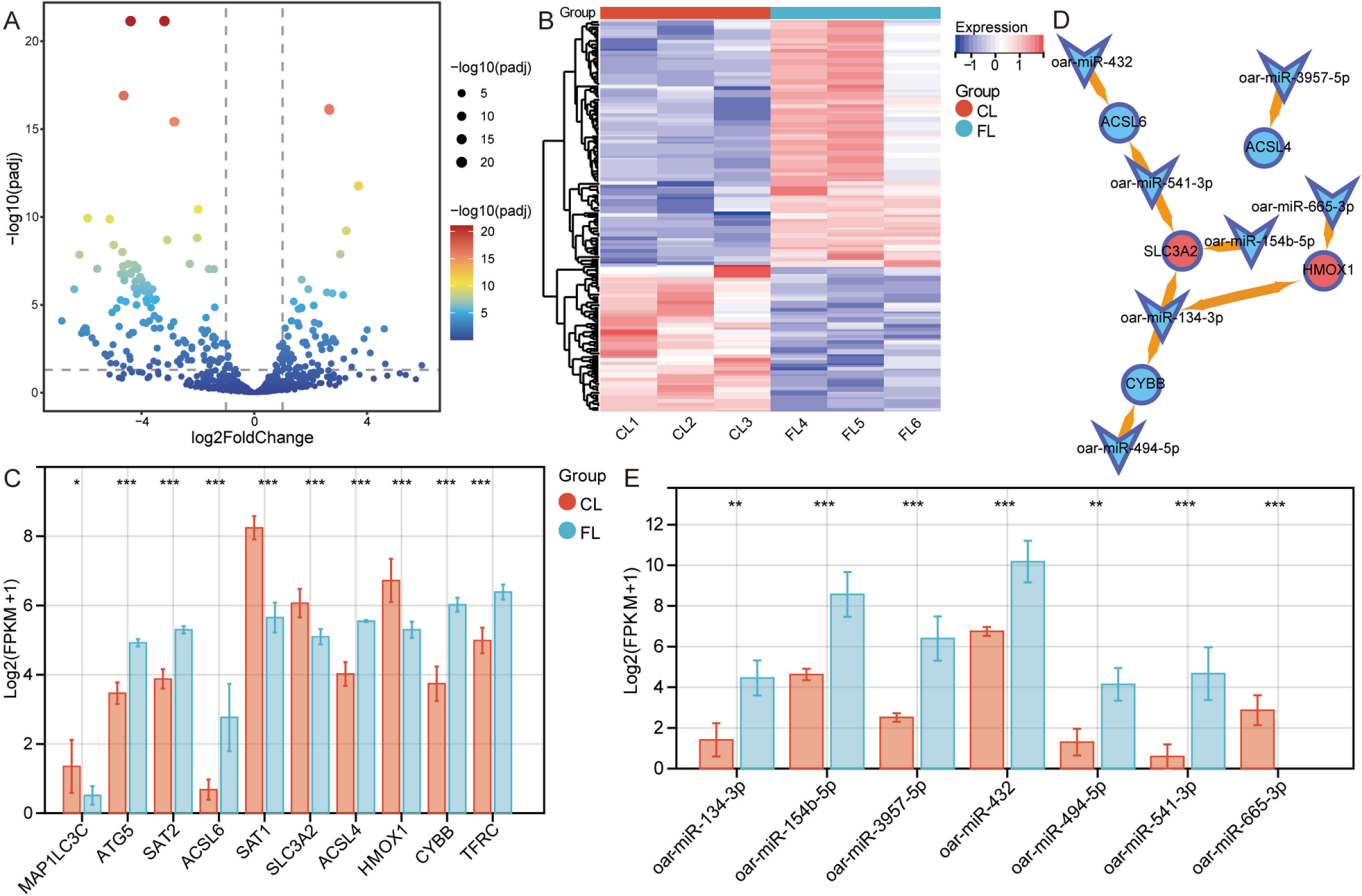
Construction of the miRNA-regulated network for ferroptosis-related genes. **A** Volcano map of differentially expressed miRNAs in GCs between CLs and FLs. **B** Heatmap of differentially expressed miRNAs in GCs between CLs and FLs. **C** Differentially expressed ferroptosis-related genes in GCs between CLs and FLs. **D** Regulatory network of DE-miRNAs to differentially expressed ferroptosis-related genes. **E** The expression of miRNAs that regulate ferroptosis-related genes in GCs between CLs and FLs. * *p* < 0.05, ** *p* < 0.01, *** *p* < 0.001

Differential expression analysis revealed 304 DE-miRNAs: 210 novel miRNAs and 94 known miRNAs (Fig. 3A, B). To identify the miRNA regulatory network of ferroptosis-related genes, we first observed the differences in ferroptosis-related genes between CLs and FLs (Fig. 3C). The miRanda database predicted that 15203 target genes may be regulated by DE-miRNAs, and the RNAhybrid database predicted that 27511 target genes may be regulated by DE-miRNAs. By comparison with the target genes, we finally identified 5 ferroptosis-related genes as target genes of known DE-miRNAs (Fig. 3D). Among them, HMOX1 and SLC3A2 were expressed at lower levels in FLs than in CLs, and the expression levels of miRNAs were all higher in FLs than in CLs (Fig. 3E). Finally, we hypothesized that HMOX1 may be regulated by oar-miR-665-3p and oar-miR-134-3p and that SLC3A2 may be regulated by oar-miR-541-3p, oar-miR-154b-5p, and oar-miR-134-3p.

### Verification of ferroptosis-related regulatory networks

**Fig. 4:**
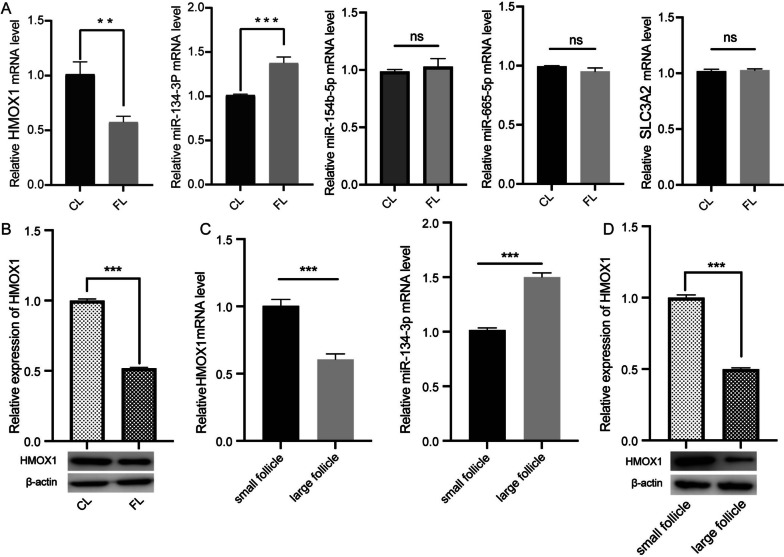
Analysis of ferroptosis-related miRNAs and mRNAs in the regulatory network. **A** The mRNA expression of genes in ferroptosis-related regulatory networks detected by RT‒qPCR in CL and FL. **B** Relative expression of HMOX1 detected by Western blot in CLs and FLs. Original blots are presented in Supplementary Fig. 2A. **C** The mRNA expression of HMOX1 and oar-miR-134-3p detected by RT‒qPCR in GCs from large follicles and small follicles. **D** Relative expression of HMOX1 detected by Western blot in GCs from large follicles and small follicles. Original blots are presented in Supplementary Fig. 2B. *P < 0.05, **P < 0.01. ns, not significant. CLs, control lambs; FLs, lambs treated with FSH

To validate the expression of the ferroptosis-related miRNA regulatory network, we conducted qRT‒PCR and Western blot experiments in CLs and FLs. The results confirmed that the mRNA level of HMOX1 was lower in FLs than in CLs, while oar-miR-134-3p was higher in FLs than in CLs (Fig. 4A). However, there was no significant difference in the levels of SLC3A2, oar-miR-541-3p, and oar-miR-154b-5p between the two groups. The protein expression of HMOX1 was also significantly lower in FLs than in CLs (Fig. 4B). Furthermore, we determined the levels of HMOX1 and miR-134-3p in GCs from large follicles and small follicles. The mRNA level of HMOX1 in large follicles was lower than that in small follicles, while the mRNA level of oar-miR-134-3p in large follicles was higher than that in small follicles (Fig. 4C). Western blot experiments also confirmed that the expression of HMOX1 was significantly lower in large follicles than in small follicles (Fig. 4D).

### oar-miR-134-3p targets HMOX1

**Fig. 5:**
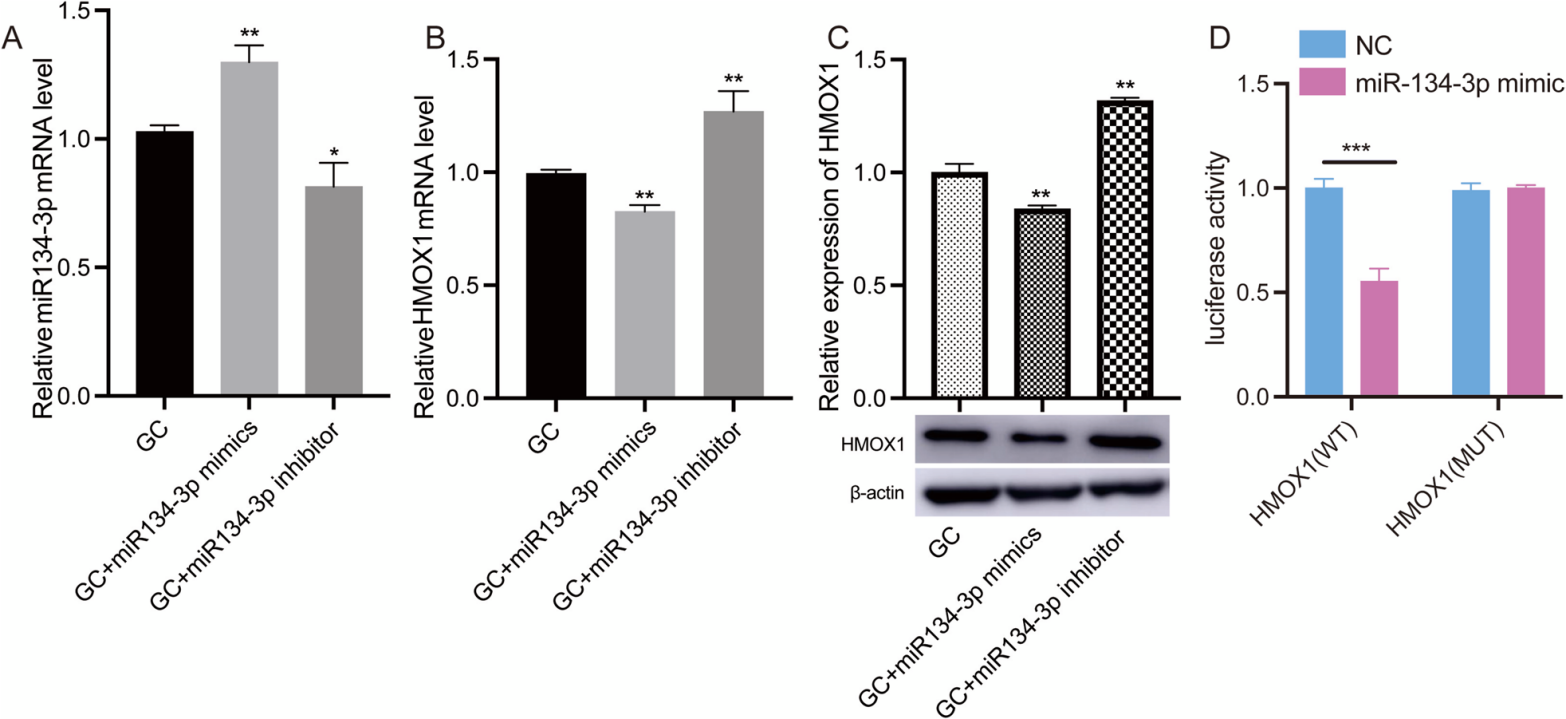
oar-miR-134-3p regulated HMOX1 expression. **A** The mRNA expression of oar-miR-134-3p determined by RT‒qPCR in GCs transfected with oar-miR-134-3p mimics, inhibitor, and NC. **B** The mRNA expression of HMOX1 determined by RT‒qPCR in GCs transfected with oar-miR-134-3p mimics, inhibitor, and NC. **C** Relative expression of HMOX1 detected by Western blot in GCs transfected with oar-miR-134-3p mimics, inhibitor, and NC. Original blots are presented in Supplementary Fig. 3. **D** Luciferase activities in cells transfected with a luciferase plasmid containing the wild-type (WT) or mutated (MUT) HMOX1 3′ UTR and either the oar-miR-134-3p mimic or a negative control (NC). *P < 0.05, **P < 0.01, ***P < 0.001

We regulated the expression of oar-miR-134-3p using GCs from large follicles. Transfection of oar-miR-134-3p mimics significantly increased the expression of oar-miR-134-3p in GCs, while transfection of oar-miR-134-3p inhibitor significantly decreased the expression of oar-miR-134-3p (Fig. 5A). Transfection of oar-miR-134-3p mimics decreased the expression of HMOX1, while transfection of the oar-miR-134-3p inhibitor resulted in an increase in HMOX1 expression (Fig. 5B, C). The dual-luciferase reporter assay showed a significant decrease in luciferase activity when WT-HMOX1 and oar-miR-134-3p mimics were cotransfected (Fig. 5D).

### oar-miR-134-3p regulates ferroptosis and affects cell proliferation

**Fig. 6:**
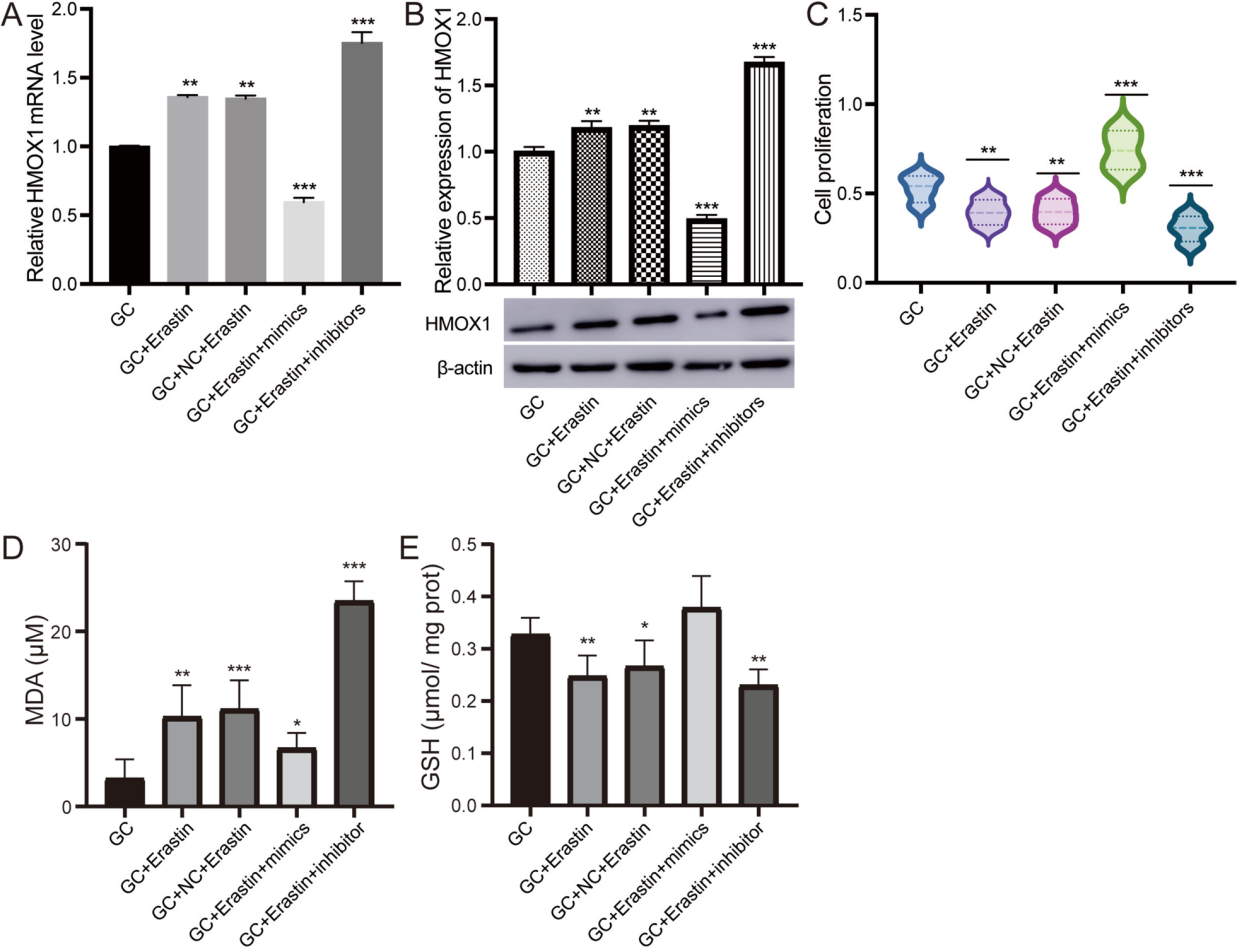
oar-miR-134-3p promotes cell proliferation by inhibiting ferroptosis. **A** The mRNA expression of HMOX1 determined by RT‒qPCR in GCs subjected to different treatments. **B** Relative expression of HMOX1 determined by Western blots in different treatments on GCs. Original blots are presented in Supplementary Fig. 4. **C** MDA levels in different treatments on GCs. **D** GSH levels in different treatments on GCs. **E** Cell proliferation measured with a CCK-8 kit in different treatment groups of GCs. **P < 0.01, ***P < 0.001. GC, granulosa cell; mimics, oar-miR-134-3p mimics; inhibitor, oar-miR-134-3p inhibitor

To further investigate the effects of ferroptosis on GCs, we first induced ferroptosis using erastin treatment for 24 h, and the expression level of HMOX1 was found to be the highest with 0.1 μM erastin (Figure S 1). Compared to the control, erastin treatment increased the expression of HMOX1 in GCs (Fig. 6A, B). When erastin-treated cells were transfected with oar-miR-134-3p mimics, the expression of HMOX1 significantly decreased, while the level of oar-miR-134-3p significantly increased. In contrast, in erastin-treated cells transfected with the oar-miR-134-3p inhibitor, the expression of HMOX1 significantly increased, while the level of oar-miR-134-3p decreased significantly.

Additionally, we observed that erastin treatment significantly reduced the proliferation of GCs, which was improved in the erastin treatment group transfected with oar-miR-134-3p mimics (Fig. 6C). Compared to that in the control group, the level of MDA released in erastin-treated GCs increased, while the level of MDA decreased in cells treated with erastin and transfected with the oar-miR-134-3p inhibitor (Fig. 6D). Conversely, the level of GSH released in erastin-treated GCs decreased, while the level of GSH increased in cells treated with erastin and transfected with the oar-miR-134-3p inhibitor (Fig. 6E).

**Fig. 7:**
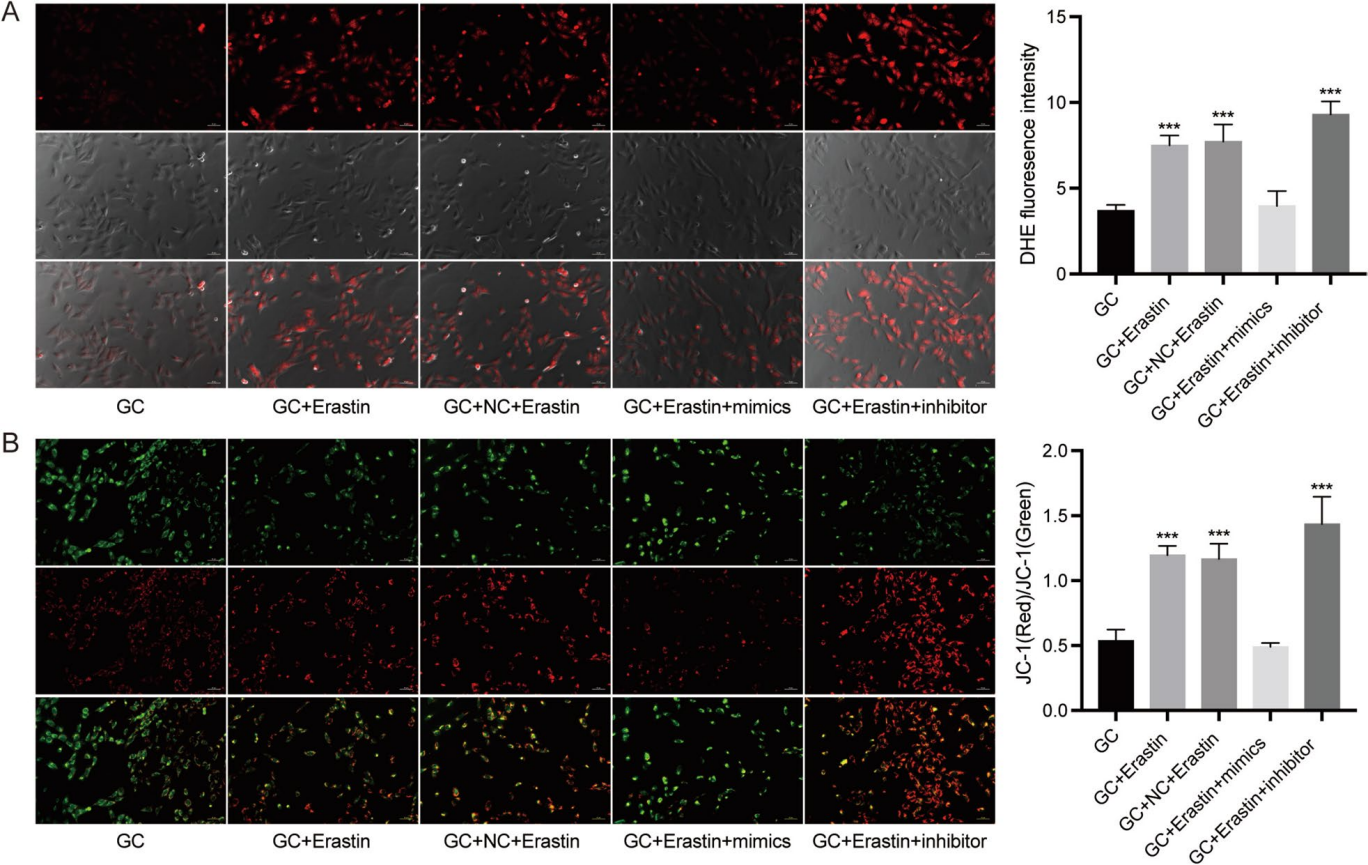
ROS and mitochondrial membrane potential regulated by ferroptosis and oar-miR-134-3p in GCs. **A** ROS production in different treatment groups of GCs stained with DHE. **B** Fluorescence image and ratio of red/green fluorescence in different treatment groups of GCs stained with JC-1. ***P < 0.001

Further study found that the levels of ROS were increased by erastin treatment or treatment with the oar-miR-134-3p inhibitor, and this effect was identified in the erastin treatment with oar-miR-134-3p mimics group (Fig. 7A). The level of mitochondrial membrane potential was also determined by JC-1 staining. The ratio of red to green fluorescence was increased in GCs treated with erastin alone or together with the oar-miR-134-3p inhibitor, aggravating this effect (Fig. 7B). In the erastin and oar-miR-134-3p mimics groups, a drop in the ratio of red to green fluorescence was notable.

## Discussion

GCs are integral to the reproductive competence of sheep, serving as the nexus for oocyte maturation and ovarian fertility [13]. A wealth of knowledge emphasizes the important role of noncoding RNAs in sheep reproduction, with miRNAs emerging as key modulators of follicular development [14, 15]. This study utilized high-throughput sequencing to analyse the gene expression landscape of GCs from Merino lambs treated with and without FSH to elucidate the molecular interplay between miRNAs and ferroptosis. The research results revealed the crucial role that ferroptosis may play in the process of follicle maturation, as well as the important regulatory role of miRNAs in this process. The advantage of this study over previous research lies in its targeted focus on GCs and the exploration of the miRNA-mediated regulation of ferroptosis in relation to cell proliferation and development.

Our findings illuminate the substantial enrichment of differentially expressed mRNAs in pathways critical to follicle development, such as hypoxia response and glycolysis, aligning with the established understanding of their roles in GC function and oocyte maturation [16–18]. Additionally, our research suggests a potential association of these genes with KEGG pathways such as ferroptosis, insulin resistance, and the cell cycle. Insulin resistance in GCs is a contributing factor to reproductive disorders in patients with polycystic ovary syndrome [19]. GCs are highly sensitive to ferroptosis, and the induction of ferroptosis may promote cell damage, resulting in poor oocyte quality [20]. These findings provide new clues for understanding the gene regulatory network involved in follicle development.

We validated the regulation of the ferroptosis-related genes HMOX1 and GSH by oar-miR-134-3p, providing new evidence for its role in follicle maturation. Studies have indicated that oar-miR-134-3p may play an important role in sheep breeding by regulating thyroid hormone synthesis [21]. Furthermore, we found that in large follicles, the level of HMOX1 was significantly lower than that in small follicles, while the expression of oar-miR-134-3p was significantly higher. The HMOX1 gene encodes haem oxygenase 1, which is a key enzyme involved in iron metabolism and the oxidative stress response and is crucial for maintaining follicle health [22, 23]. GSH depletion is considered a fundamental characteristic of ferroptosis [24]. Research has shown that a decrease in GSH levels promotes ferroptosis in GCs [25]. In our study investigating the regulation of oar-miR-134-3p expression, we discovered its significant negative regulation of HMOX1 expression. This finding reveals the crucial role of oar-miR-134-3p in regulating the ferroptosis process in GCs. HMOX1’s modulation by oar-miR-134-3p highlights a miRNA-mediated target in ferroptosis regulation within GCs.

Furthermore, we found that erastin-induced ferroptosis affects the proliferation level of GCs, and this effect can be regulated by oar-miR-134-3p. This finding not only corroborates the susceptibility of GCs to ferroptosis but also reveals a potential therapeutic avenue to modulate this cell death pathway. Additionally, our study revealed that erastin treatment affects ROS levels and mitochondrial membrane potential, both of which play important roles in cellular stress responses and apoptosis processes [26–28]. These findings are consistent with previous research indicating that iron overload and oxidative stress are important regulatory factors in follicle development [29]. Ferroptosis, a form of iron and ROS-dependent regulated cell death, has been found to involve the accumulation of lipid peroxidation products and lethal ROS during ferroptosis [30]. HMOX1 has antioxidant and antiapoptotic effects in GCs [31]. In addition to the consumption of GSH, erastin can induce ferroptosis by enhancing the expression of HMOX1 as a nonclassical mode [22]. The impact of oar-miR-134-3p on these cellular physiological processes further emphasizes its crucial role in ferroptosis and follicle maturation through targeting HMOX1.

This study has some limitations. The scope of our experimental validation was confined to ferroptosis-related mRNAs and miRNAs, potentially overlooking other critical genes in GC development. Moreover, our research mainly focused on the regulatory mechanisms of gene expression, with limited exploration of specific cellular signalling pathways and molecular mechanisms. Further experimental studies are needed to elucidate how miRNAs precisely regulate target genes and how these genes influence GC function and ferroptosis. Additionally, while we observed the impact of erastin-induced ferroptosis on GCs, this effect may vary at different stages of follicle development.

## Conclusion

In summary, our study reveals the regulatory mechanism between oar-miR-134-3p and ferroptosis-related genes, demonstrating its presence in GCs. This finding provides a theoretical basis and experimental evidence for further research on the ferroptotic mechanism in GCs and potential therapeutic strategies for related reproductive disorders.

### Supplementary Information

**Additional file 1:** **Table S1.** Primer sequences.

**Additional file 2:** **Figure S1.** Relative expression of HMOX1 determined by Western blot analysis in GCs treated with erastin. **Figure S2.** Images of expression of HMOX1 detected by Western blot. **Figure S3.** Images of expression of HMOX1 detected by Western blot in GCs transfected with oar-miR-134-3p mimics, inhibitor, and NC. **Figure S4.** Images of expression of HMOX1 detected by Western blot in different treatments on GCs.

## Data Availability

The datasets generated and/or analyzed during the current study are available from corresponding author.
