# Supplementary material for: MiR-134-3p targets HMOX1 to inhibit ferroptosis in granulosa cells of sheep follicles
Source: J Ovarian Res. 2024 Jan 2;17:3. doi: 10.1186/s13048-023-01328-6 (PMC10763389; doi:10.1186/s13048-023-01328-6)
Supplement: Supplementary file 1 — Additional file 1: Table S1. Primer sequences. [file 13048_2023_1328_MOESM1_ESM.docx]

Table S1. Primer sequences.

| Gene | Primer sequences |
| --- | --- |
| oar-miR-134-3p | F: 5′- CGTCTGGGCTGCCTCGTC -3′ |
|  | R: 5′- TGGTGTCGTGGAGTCG -3′ |
| oar-miR-154b-5p | F: 5′- GCGCGATCATACATGGTTGAC -3′ |
|  | R: 5′- GTGCAGGGTCCGAGGT -3′ |
| oar-miR-665-3p | F: 5′- GCGAGGGGTCTTGGCCT -3′ |
|  | R: 5′- CTCAACTGGTGTCGTGGA -3′ |
| oar-miR-541-3p | F: 5′- GCGTGGTGGGCACAGAA -3′ |
|  | R: 5′- GACACAGGTGCCATCGGAAA -3′ |
| oar-U6 | F: 5′- AACGCTTCACGAATTTGCGT -3′ |
|  | R: 5′- GTTGCGCTTCACAACTCAGG-3′ |
| oar-HMOX1 | F: 5′- AGAGTTCTCGGAGCCAGCAT -3′ |
|  | R: 5′- ATGAACTCGGCATTCTCCGC -3′ |
| aor-SLC3A2 | F: 5′- CCCCAGTGTTCAGCTATGGAG -3′ |
|  | R: 5′- AGGTGTTGGGAAAGCTGGATT -3′ |
| B2M | F: 5′- CACAACCCAAGATAGTTAAGTGGG -3′ |
|  | R: 5′- GGGCCCAAGGTAGAATTATAAAGAA -3′ |
