# Supplementary material for: MiR-134-3p targets HMOX1 to inhibit ferroptosis in granulosa cells of sheep follicles
Source: J Ovarian Res. 2024 Jan 2;17:3. doi: 10.1186/s13048-023-01328-6 (PMC10763389; doi:10.1186/s13048-023-01328-6)
Supplement: Supplementary file 2 — Additional file 2: Figure S1. Relative expression of HMOX1 determined by Western blot analysis in GCs treated with erastin. Figure S2. Images of expression of HMOX1 detected by Western blot. Figure S3. Images of expression of HMOX1 detected by Western blot in GCs transfected with oar-miR-134-3p mimics, inhibitor, and NC. Figure S4. Images of expression of HMOX1 detected by Western blot in different treatments on GCs. [file 13048_2023_1328_MOESM2_ESM.pdf]

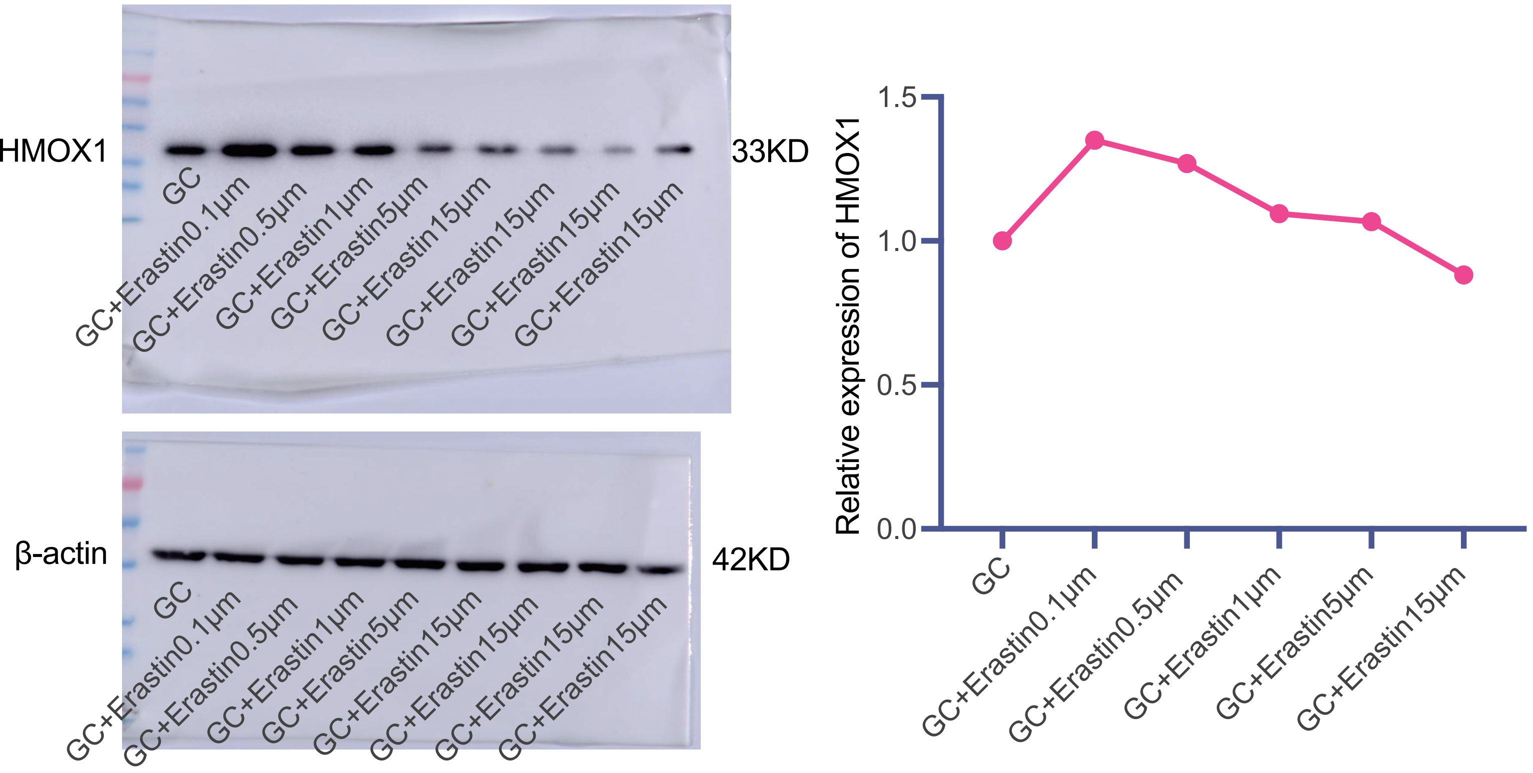


Figure S1. Relative expression of HMOX1 determined by Western blot analysis in GCs treated with erastin.

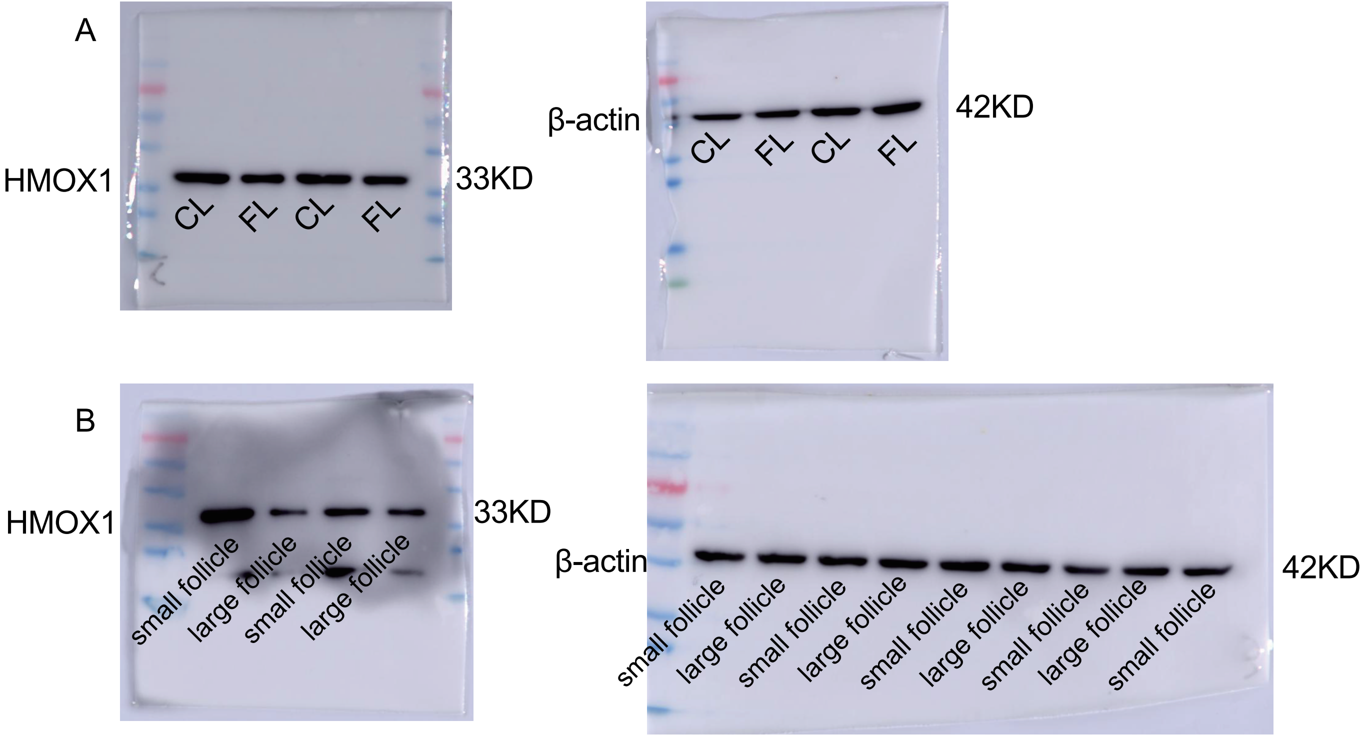


Figure S2. Images of expression of HMOX1 detected by Western blot.

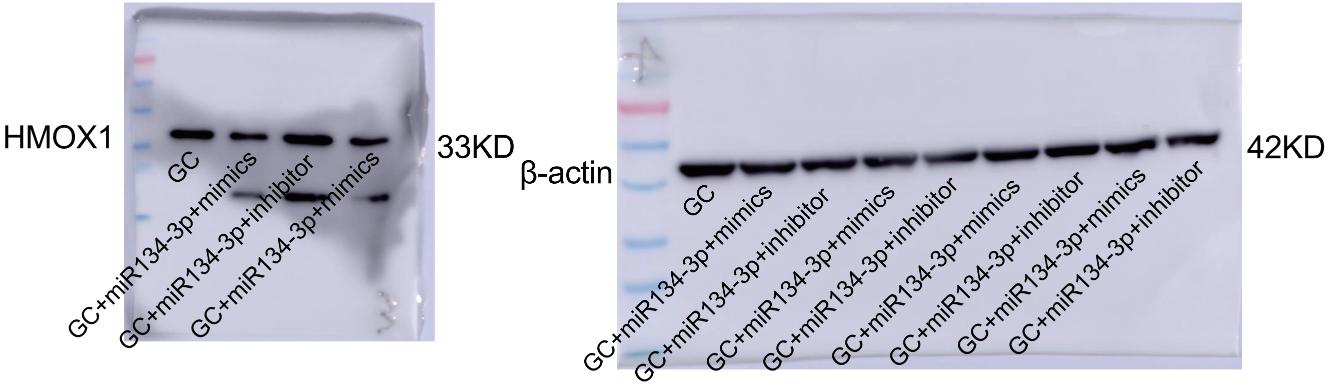


Figure S3. Images of expression of HMOX1 detected by Western blot in GCs transfected with oar-miR-134-3p mimics, inhibitor, and NC.

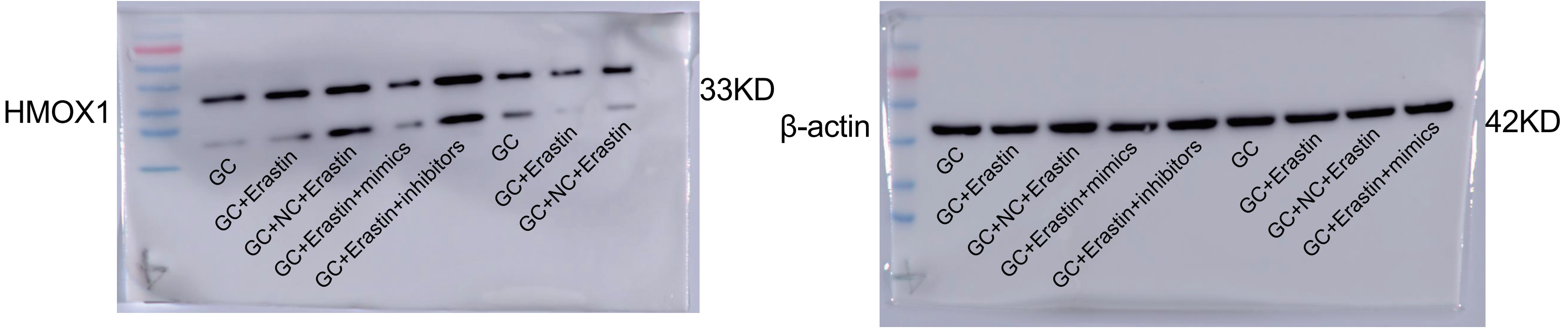


Figure S4. Images of expression of HMOX1 detected by Western blot in different treatments on GCs.
